# The complete chloroplast genome sequence of *Populus wilsonii* based on landscape design, and a comparative analysis with other *Populus* species

**DOI:** 10.1080/23802359.2020.1787901

**Published:** 2020-07-09

**Authors:** Sen Du

**Affiliations:** School of Architecture and Urban Planning, Lanzhou Jiaotong University, lanzhou, China

**Keywords:** Landscape design, *P. wilsonii*, chloroplast genome, phylogenetic analysis

## Abstract

In the landscape planning and design, the ecological concept should occupy a dominant position, and the construction of an ‘environmentally friendly, resource-saving’ ecological new city is inseparable from the cultivation of forest tree species. *Populus wilsonii*, is an important landscape design tree species. *Populus wilsonii*, is also an important ecological role in boreal and temperate forests, serving as wildlife habitats and watersheds and is an important landscape ornamental tree species. The complete chloroplast genome sequence of *Populus wilsonii* was characterized from Illumina pair-end sequencing. The chloroplast genome of *P. wilsonii* was 155,716 bp in length, containing a large single-copy region (LSC) of 85,022 bp, a small single-copy region (SSC) of 16,626 bp, and two inverted repeat (IR) regions of 27,034 bp. The overall GC content is 36.70%, while the correponding values of the LSC, SSC, and IR regions are 34.5%, 30.6%, and 42.0%, respectively. The genome contains 129 complete genes, including 84 protein-coding genes (62 protein-coding gene species), 37 tRNA genes (29 tRNA species) and 8 rRNA genes (4 rRNA species). The Neighbour-joining phylogenetic analysis showed that *P. wilsonii* and *Populus cathayana* clustered together as sisters to other *Populus* species.

## Introduction

The principle of natural ecology is from the perspective of the esthetic function and practical function of the landscape. In terms of esthetics, the most brilliant landscaping should reach the state of ‘although it is made by humans, just like nature’, so ‘natural’ is the ultimate in landscape design aims. ‘Ecology’ refers to treating a piece of land to be developed, only fully respecting the land's topography and landform, ‘following the mountain’, rational planning, to balance the energy conversion of land, animals and plants, microorganisms, sunlight, rain and dew with human energy. Only when people and nature live in harmony can we create a sustainable landscape. In the landscape planning and design, the ecological concept should occupy a dominant position, and the construction of an ‘environmentally friendly, resource-saving’ ecological new city is inseparable from the cultivation of forest tree species. In plant configuration, full consideration should be given to biodiversity, with native plants as the main body, as much as possible to simulate the natural increase of plant communities and the introduction of excellent new plant varieties, reasonable allocation of trees, shrubs, vines and herbs Stable multi-level and multi-structure plant communities protect the stability of park ecosystems and green landscapes. This is particularly important for the configuration of road landscape green belts and park greenery plants. *Populus wilsonii*, is an important landscape design tree species. *Populus wilsonii* occurs in northern and central parts of China, plus Mongolia, Korea, and the Far East of Russia. *P. wilsonii* is widely distributed in the Northern Hemisphere and plays an important ecological role in boreal and temperate forests, serving as wildlife habitats and watersheds; they can dominate riparian forests, but are ecologically adaptable. *P. wilsonii* has wide geographic distribution, high intraspecific polymorphism, adaptability to different environments, combined with a relatively small genome size. Consequently, *P. wilsonii* represents an excellent model for understanding how different evolutionary forces have sculpted the variation patterns in the genome during the process of population differentiation and ecological speciation (Neale and Antoine 2011). Moreover, we can develop conservation strategies easily when we understand the genetic information of *P. wilsonii*. In the present research, we constructed the whole chloroplast genome of *P. wilsonii* and understood many genome varition information about the species, which will provide beneficial help for population genetics studies of *P. wilsonii.*

The fresh leaves of *P. wilsonii* were collected from Lijiang city (100°23'N, 26°88'E). Fresh leaves were silica-dried and taken to the laboratory until DNA extraction. The voucher specimen (YY001) was laid in the Herbarium of Lanzhou Jiaotong University and the extracted DNA was stored in the −80 °C refrigerator of the School of Architecture and Urban Planning. We extracted total genomic DNA from 25 mg silica-gel-dried leaf using a modified CTAB method (Doyle 1987). The whole-genome sequencing was then conducted by Biodata Biotechnologies Inc. (Hefei, China) with Illumina Hiseq platform. The Illumina HiSeq 2000 platform (Illumina,San Diego, CA) was used to perform the genome sequence. We used the software MITObim 1.8 (Hahn et al. 2013) and metaSPAdes (Nurk et al. 2017) to assemble chloroplast genomes. We used *P. tremula* (GenBank: NC_027425) as a reference genome. We annotated the chloroplast genome with the software DOGMA (Wyman et al. 2004), and then corrected the results using Geneious 8.0.2 (Campos et al. 2016) and Sequin 15.50 (http://www.ncbi.nlm.nih.gov/Sequin/).

The complete chloroplast genome of *P. wilsonii* (GenBank accession number MT482541) was characterized from Illumina pair-end sequencing. The chloroplast genome of *P. wilsonii* was 155,716 bp in length, containing a large single-copy region (LSC) of 85,022 bp, a small single-copy region (SSC) of 16,626 bp, and two inverted repeat (IR) regions of 27,034 bp. The overall GC content is 36.70%, while the correponding values of the LSC, SSC, and IR regions are 34.5%, 30.6%, and 42.0%, respectively. The genome contains 129 complete genes, including 84 protein-coding genes (62 protein-coding gene species), 37 tRNA genes (29 tRNA species) and 8 rRNA genes (4 rRNA species).

**Figure 1.**
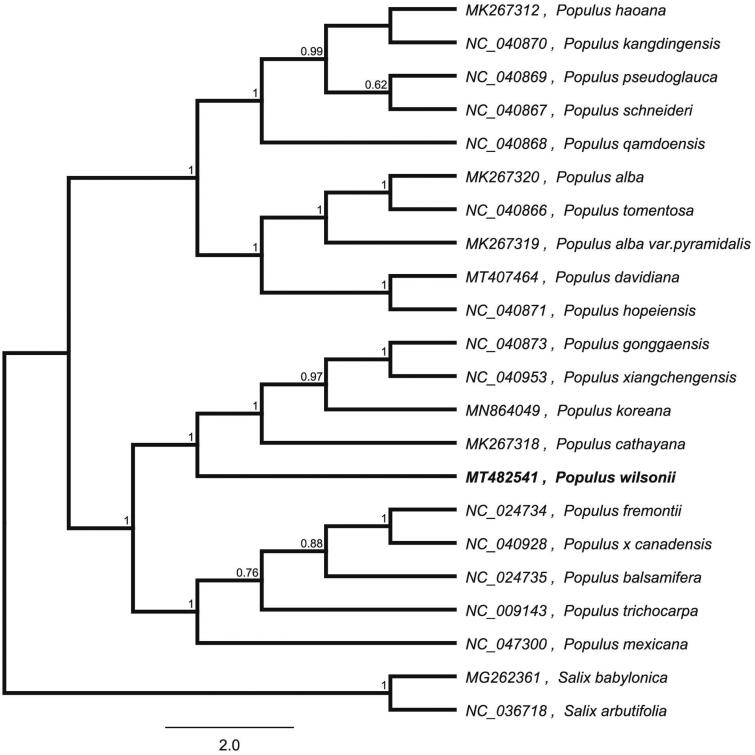
Neighbour-joining (NJ) analysis of *P. wilsonii* and other related species based on the complete chloroplast genome sequence.

To confirm the phylogenetic location of *P. wilsonii* within the family of *Populus*, we used the complete chloroplast genomes sequence of *P. wilsonii* and 21 other related species of *Populus* and *Salix babylonica* and *Salix arbutifolia* as outgroup to construct phylogenetic tree. The 22 chloroplast genome sequences were aligned with MAFFT (Katoh and Standley 2013), and then the Neighbour-joining tree was constructed by MEGA 7.0 (Kumar et al. 2016). The results confirmed that *P. wilsonii* was clustered with *Populus cathayana* (Figure 1).

## Data Availability

The data that support the findings of this study are openly available in National Center for Biotechnology Information (NCBI) at https://www.ncbi.nlm.nih.gov, accession number MT482541.
